# Liver transplantation in patients with history of extra-hepatic malignancies

**DOI:** 10.3389/frtra.2026.1723729

**Published:** 2026-03-05

**Authors:** Jerry Xiao, Ashton A. Connor, Ahmed Elaileh, Khush Patel, Samar Semaan, Youssef Dib, Jason Todd, Linda W. Moore, Sudha Kodali, David W. Victor III, Maen Abdelrahim, Anaum Maqsood, Caroline J. Simon, Yee Lee Cheah, Constance M. Mobley, Ashish Saharia, A. Osama Gaber, R. Mark Ghobrial, Kirk Heyne

**Affiliations:** 1 Department of Surgery, Houston Methodist Hospital, Houston, TX, United States; 2 Department of Medicine, Houston Methodist Hospital, Houston, TX, United States

**Keywords:** extrahepatic malignancy, liver transplant, post-transplant malignancy, pre-transplant malignancy, recurrence

## Abstract

**Introduction:**

Increasing use of solid organ transplantation [SOT] has coincided with increasing cancer survivorship. Consensus statements exist for SOT in patients with pre-transplant malignancy [PTM]. Yet, most outcomes have been reported in heart and kidney transplant. This paper addresses the shortage of information on liver transplant [LT] in patients with PTM.

**Methods:**

A retrospective case-control study was conducted of patients who underwent LT between 1/1/2008–5/31/2024 at an American transplant center. Patients were stratified according to history of extrahepatic PTM, time from PTM to LT, and post-LT PTM recurrence. Primary outcomes were overall survival [OS] and time to recurrence.

**Results:**

1,876 patients underwent LT. 143(7.62%) had an extrahepatic PTM pre-LT. PTM patients were older and had lower MELD at LT. There was no significant difference in post-LT survival (*p* = 0.293) between patients who did and did not have PTM. Of 121 patients with known time from PTM to LT, 19(15.7%) had an interval less than 2 years. When stratifying by 2-year interval from PTM to LT, there was no difference in survival (*p* = 0.34). Post-LT, 20 patients (14.0%) had recurrence of their PTM. The average time to recurrence was 595.5 days. When treated as a time-dependent co-variate, recurrence was a strong predictor of worse post-LT survival (HR 10.9, 95% CI 4.32–27.7, *p* < 0.001).

**Conclusion:**

In our experience**,** a history of pre-LT PTM, including with an interval to LT of less than 2 years, was not associated with worse post-LT survival. Recurrence of PTM did portend worse prognosis.

## 1 Introduction

In 2023, there were 172,409 solid organ transplantations [SOTs] performed throughout the world, representing a 10% increase over the year before (1). In the United States [U.S.] alone, 2024 liver transplants climbed by 8% to 11,458 procedures (2). The number of cancer survivors living 5 or more years has also grown. In 1975, only 49% lived this long, but by 2019 it was 69%. For breast and prostate cancer, as of 2019, more than 90% will live 5 or more years (3, 4).

It is not surprising that these two populations should increasingly overlap. Kidney transplants [KT], comprising nearly two thirds of all SOT, report pre-transplant malignancies [PTM] in 8% of their patients in recent years (5). Heart transplantation [HT], comprising 6% of SOTs, reports a similar percentage of 8% (6). Liver transplantation [LT], despite comprising nearly a quarter of organ transplants, has scant literature describing this overlap. This can be partially explained by LT's unique role as curative therapy for hepatic neoplasms in addition to organ replacement.

We report here our experience at a single high-volume center in the hopes of adding substantially to the present understanding of PTMs in LT. We focus on outcomes of patients with and without pre-LT PTM, with intervals from PTM to LT of lesser or greater than 2 years, and with post-LT PTM recurrence.

## 2 Materials and methods

This is a retrospective case-control study of adult patients who underwent LT at a single, American institution between 1/1/2008–5/31/2024. All research was conducted in accordance with both the Declarations of Helsinki and Istanbul. All research was approved by the Institutional Review Board of the Houston Methodist Research Institute (Protocol number 00000587:1; 4/23/2007). Written consent was given in writing by all subjects. Data was abstracted from the Organ Procurement and Transplantation Network and the institutional electronic medical record. Patients with a history of extrahepatic PTM were identified. No LT patients were excluded from this study.

Data collected included extrahepatic PTM anatomic site, time from PTM to transplant, recurrence of PTM, and time from transplant to recurrence. PTM histopathology was not available for most patients and thus not included in this study. PTM were categorized according to anatomic location (brain, breast, colorectal, cutaneous, esophageal, gastrointestinal [GI], genitourinary [GU], gynecologic [GYN], head/neck, hematologic [HEME], lung, and thyroid). The cutaneous malignancy category included basal cell, squamous cell, melanoma and other less common skin cancer histologies. Patients with a history of primary hepatobiliary [HB] neoplasms alone were stratified to the no-PTM group. Patients with both a HB neoplasm and extrahepatic PTM were stratified to the PTM group. Recurrence of PTM was defined as post-transplant malignancy [post-TM] arising from the same anatomic site. De-novo, post-TM were not examined in this study. Date of PTM and date of recurrence were defined as the first mention of the malignancy in the electronic medical record. Primary outcomes were overall survival [OS] from date of index LT. Secondary outcomes were time from index LT to recurrence of PTM. Patients with missing data were not included in the respective subgroup analyses requiring such data.

Peri-LT continuous and categorical variables were compared using appropriate parametric and non-parametric tests. Associations with time-to-event outcomes, including post-LT OS and time to post-LT PTM recurrence, were determined using uni- and multi-variable Cox proportional hazards models. To determine its association with post-LT OS, time to PTM recurrence was treated as a time-dependent co-variable.

At the study institution, LT evaluation mandates age-appropriate screening for occult malignancies and review at multidisciplinary transplant medical board. Patients with a history of PTM are only deemed candidates for LT if their PTM had been treated with curative intent, is in complete remission and/or if their survival is estimated to exceed 80% at 5 years, as estimated by the transplant team which includes a medical oncologist*.* Immunosuppression regimens follow organ-dependent institutional clinical practice guidelines. For liver-only or simultaneous liver and heart transplantation, there is no induction agent used, corticosteroids are given intra-operatively then tapered off, and maintenance therapy consists of tacrolimus (FK goal 5–9 ng/mL) and mycophenolate. For simultaneous liver and kidney transplantation, there is no induction, corticosteroids are given intra-operatively then tapered to prednisone 5 mg once daily, along with tacrolimus (FK goal 5–9 ng/mL) and mycophenolate maintenance therapy. For simultaneous liver and lung transplantation, basiliximab is used for induction, corticosteroids are tapered to prednisone 5 mg daily, along with tacrolimus (FK goal 5–9 ng/mL) and mycophenolate maintenance therapy. In patients who develop cancer recurrence following transplantation, immunosuppressive regimens and drug level goals are not usually changed.

## 3 Results

### 3.1 Patient demographics

During the study period, 1,876 patients underwent LT. Of these, 143 (7.6%) patients were identified as having a total of 159 extrahepatic PTM. The most common PTM anatomic classes identified were cutaneous (50), GU (30), colorectal (21), breast (19), GYN (12) and HEME (9) (Table 1). There were 127 patients (88.8%) with one PTM and 16 (11.2%) with two PTM's. Overall, patients were predominantly male (*n* = 1,123, 59.9%) with a median age of 58 years (IQR 49–65 years) and median MELD score at LT of 28 (IQR 16–37) (Table 2). There were 607 patients (32.4%) with HB malignancies. There were 807 (43%) patients transplanted from the intensive care unit [ICU], and 291 **(**15.5%) patients underwent simultaneous organ transplantation. Median post-LT follow-up was 1,091 days (IQR 365–2,192 days). Post-LT OS at 1, 3 and 5 years was 92.2%, 82.7%, and 76.2%, respectively.

**Table 1:** Tabulation of 159 pre-transplant extrahepatic malignancies in 143 patients by anatomical sites, including numbers of patients per tumor, intervals to liver transplant, and numbers of patients with recurrences.

Pre-transplant tumor type	Number of patients	Missing date	<2 years before transplant	>2 years before transplant	Recurrence
Cutaneous	50	15	7	28	14
Genitourinary	30	2	4	24	2
Colorectal	21	1	2	18	3
Breast	19	0	5	14	2
Gynecologic	12	2	1	9	1
Hematologic	9	1	3	5	1
Thyroid	5	0	0	5	0
Head and neck	5	0	1	4	1
Lung	3	0	1	2	0
Gastrointestinal	2	0	1	1	0
Brain	2	1	0	1	0
Esophageal	1	0	1	0	0

**Table 2:** Peri-operative liver transplant variables in patients with and without by pre-transplant extrahepatic malignancy. PTM pre-transplant malignancy; ICU intensive care unit, IQR inter-quartile range.

Variable	All patients (*n* = 1,876)	No history of PTM (*n* = 1,733)	History of PTM (*n* = 143)	*p*
Gender				
Female	753 (40.14%)	688 (39.7%)	65 (45.5%)	0.207
Male	1,123 (59.86%)	1,045 (60.3%)	78 (54.5%)	
Race/Ethnicity				0.011
Asian	68 (3.63%)	60 (3.5%)	8 (5.6%)	
Black	174 (9.29%)	168 (9.7%)	6 (4.2%)	
Hispanic	426 (22.76%)	404 (23.4%)	22 (15.4%)	
Native	7 (0.37%)	7 (0.4%)	0 (0%)	
White	1,197 (63.94%)	1,090 (63%)	107 (74.8%)	
Waitlist time (days) Median (IQR)	93.50 (10.00 to 372.50)	81.00 (9.00 to 367.00)	216.00 (41.00 to 458.00)	
Age at transplant (years) Median (IQR)	58.00 (49.00–65.00)	58.00 (48.00–64.00)	62.00 (57.00–67.00)	<.001
BMI at transplant (kg/m2) Median (IQR)	28.36 (24.60–32.93)	28.35 (24.53–32.96)	28.36 (25.40–32.78)	0.51
Condition				
Home	770 (41.04%)	688 (39.7%)	82 (57.3%)	<.001
Inpatient non-ICU	299 (15.94%)	278 (16%)	21 (14.7%)	
ICU	807 (43.02%)	767 (44.3%)	40 (28%)	
Diabetic status				
Type 1, type 2, unknown type	578 (30.81%)	517 (30%)	61 (42.7%)	0.002
No	1,290 (69.06%)	1,208 (70%)	82 (57.3%)	
History of alcohol use				0.002
No	1,248 (66.52%)	1,136 (65.6%)	112 (78.3%)	
Yes	628 (33.48%)	597 (34.4%)	31 (21.7%)	
MELD	28.00 (16.00–37.00)	28.00 (16.00–37.00)	22.00 (11.00–31.00)	<.001
Hepatitis C virus				
Negative	1,420 (77.60%)	1,318 (77.9%)	102 (73.9%)	0.331
Ab or NAT positive	410 (22.40%)	374 (22.1%)	36 (26.1%)	
Hepatitis B virus				
negative	1,664 (89.85%)	1,541 (90%)	123 (88.5%)	0.685
Ab, Ag or NAT positive	188 (10.15%)	172 (10%)	16 (11.5%)	
Etiology of liver failure				
Auto-immune	156 (8.32%)	145 (8.4%)	11 (7.7%)	0.014
Congenital	104 (5.54%)	96 (5.5%)	8 (5.6%)	
Metabolic	324 (17.27%)	295 (17%)	29 (20.3%)	
Other	266 (14.18%)	235 (13.6%)	31 (21.7%)	
Toxic (alcohol, acetaminophen)	636 (33.90%)	605 (34.9%)	31 (21.7%)	
Viral	390 (20.79%)	357 (20.6%)	33 (23.1%)	
Organ(s) transplanted				<.001
Liver only	1,585 (84.49%)	1,468 (84.7%)	117 (81.8%)	
Liver + heart	47 (2.51%)	45 (2.6%)	2 (1.4%)	
Liver + heart + kidney	4 (0.21%)	1 (0.1%)	3 (2.1%)	
Liver + heart + lung	1 (0.05%)	1 (0.1%)	0 (0%)	
Liver + kidney	219 (11.67%)	200 (11.5%)	19 (13.3%)	
liver + lung	20 (1.07%)	18 (1%)	2 (1.4%)	
Overall survival (days) Median (IQR)	1,091.00 (365.00–2,192.00)	1,093.00 (365.00–2,210.00)	735.00 (360.50–2,184.00)	
Graft Survival (days) Meidan (IQR)	1,076.50 (362.00–2,188.00)	1,082.00 (363.00–2,189.00)	730.00 (324.00–2,170.50)	
Cause of death				
Cancer	47 (13.82%)	39 (12.6%)	8 (26.7%)	0.094
Not cancer	366 (66.18%)	271 (87.40%)	22 (73.30%)	
Hepatobiliary malignancy				<.001
No	1,269 (67.64%)	1,195 (69.00%)	74 (51.70%)	
Yes	607 (32.36%)	537 (31.00%)	69 (48.30%)	

### 3.2 Comparison of patients with and without PTM

At the time of LT, patients with PTM were significantly older (median 62 vs. 58 years, *p* < 0.001), more frequently Caucasian (74.8% vs. 63%, *p* = 0.011), and more frequently had diabetes mellitus (42.7% vs. 31.94%, *p* = 0.005) (Table 2). Fewer patients with PTM reported a history of alcohol use prior to LT (21.7% vs. 34.4%, *p* < 0.001). A significantly greater proportion of patients without PTM were transplanted due to alcohol and acetaminophen-related toxicity compared to patients with a history of PTM (34.9% vs. 21.7%; *p* = 0.014). The incidence of HBV and HCV were comparable between the two groups. LT patient with PTM also had HB malignancies significantly more frequently (48.3% vs. 21%, *p* < 0.001). Waitlist time was longer in patients with PTM (216 vs. 81 days).

**Figure 1:**
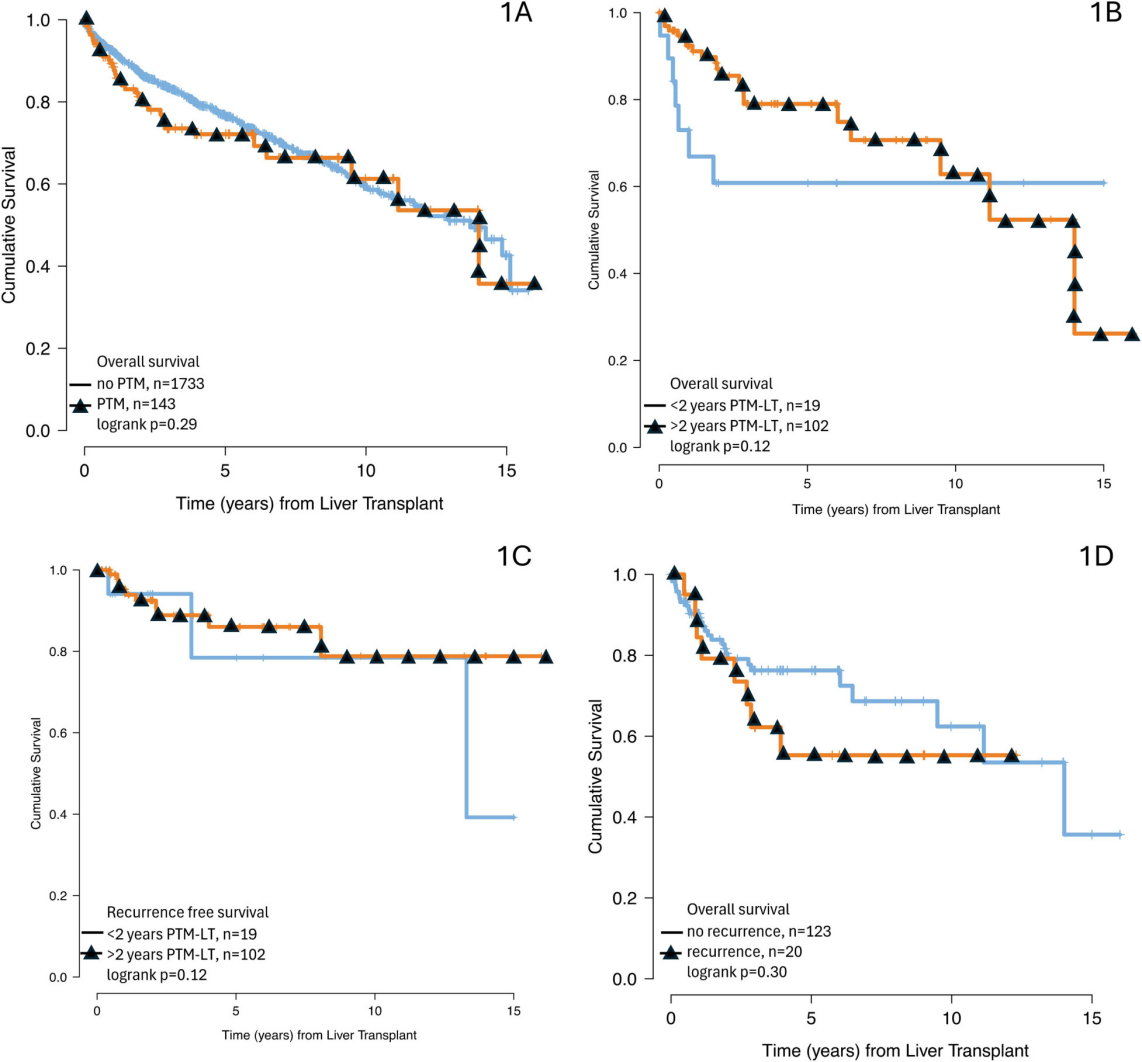
Post-transplant survival stratified by **(A)** presence of pre-transplant extrahepatic malignancy, **(B)** 2-year interval from pre-transplant malignancy to transplant, and **(D)** post-transplant recurrence of extrahepatic malignancy. Also, **(C)** post-transplant recurrence free survival stratified by 2-year interval from pre-transplant malignancy to transplant.

Patients with PTM had lower MELD scores at time of LT (median 22 vs. 28, *p* < 0.001) and presented for transplant from lower acuity settings (57.3% vs. 39.7% from home, 28% vs. 44.3% from the ICU, *p* < 0.001). There was no significant difference in post-LT OS of patients with and without PTM (HR 1.20, 95%CI 0.85–1.69, *p* = 0.293, Figure 1A; Supplementary Table S1A). There was no significant difference in recorded causes of death in patients with and without PTM (*p* = 0.09). Also, no associations were found between distinct PTM anatomic types and post-LT survival. Post-transplant survival was associated with recipient age, recipient MELD at transplant, and donor Kidney Donor Profile Index [KDPI].

### 3.3 Comparison of interval from PTM to LT

In the PTM cohort, 22 patients (15.4%) were missing date of PTM. Time from PTM to LT was available in 121 (84.6%) patients (Table 3). Median time from PTM to transplant was 2,256 days (IQR 1,174–5,614 days). In 102 (84.3%) patients, this time was greater than 2 years, with median time from PTM to transplant of 3,165 days (IQR 1,763–5,829 days). In these patients, the most common PTM categories were cutaneous, GU, and colorectal. For 19 (15.7%) patients, time from PTM to transplant was less than 2 years, with median time of 324 days (IQR 142.5–397.5 days). In these patients, the most common PTM categories were cutaneous, breast, and GU. No significant differences were identified in PTM anatomic classes nor in patient variables between these two groups. Similarly, there was no significant association of post-LT OS with interval from PTM to LT (HR 0.51, 95%CI 0.21–1.21, *p* = 0.126, Figure 1B; Supplementary Table S1B). The number of and time to post-LT PTM recurrences were also not significantly associated with time from malignancy to transplant (Table 3; Figure 1C).

**Table 3:** Peri-operative liver transplant variables in patients with less than or greater than 2 year interval from pre-transplant extrahepatic malignancy to liver transplant in 121 patients with known intervals.

Variable	All patients (*n* = 121)	<2 years between PTM and transplant (*n* = 19)	>2 years between PTM and transplant (*n* = 102)	*p*
Time from PTM to transplant (days) Median (IQR)	2,256.00 (1,174.00–5,614.00)	324.00 (142.50–397.50)	3,164.50 (1,763.00–5,829.00)	
Gender				
Female	65 (45.45%)	12 (63.2%)	45 (44.1%)	0.202
Male	78 (54.55%)	7 (36.8%)	57 (55.9%)	
Waitlist time (days) Median (IQR)	216.00 (41.00–458.00)	211.00 (59.00–274.50)	250.50 (70.00–492.00)	
Age at transplant (years) Median (IQR)	62.00 (57.00–67.00)	60.00 (56.00–67.00)	62.00 (57.00–67.00)	0.786
BMI (kg/m^2^) Median (IQR)	28.36 (25.40–32.78)	29.45 (24.96–32.30)	28.05 (25.68–33.05)	0.912
Condition				
Home	82 (57.34%)	9 (47.4%)	63 (61.8%)	0.49
Inpatient non-ICU	21 (14.69%)	3 (15.8%)	13 (12.7%)	
ICU	40 (27.97%)	7 (36.8%)	26 (25.5%)	
Etiology of liver failure				
Auto-immune	11 (7.69%)	0 (0%)	6 (5.9%)	0.422
congenital	8 (5.59%)	1 (5.3%)	5 (4.9%)	
Metabolic	29 (20.28%)	3 (15.8%)	24 (23.5%)	
Other	31 (21.68%)	4 (21.1%)	24 (23.5%)	
Toxic	31 (21.68%)	3 (15.8%)	22 (21.6%)	
Viral	33 (23.08%)	8 (42.1%)	21 (20.6%)	
MELD at transplant	22.00 (11.00–31.00)	27.00 (11.50–36.00)	21.00 (11.00–30.00)	0.213
Overall survival (days) Median (IQR)	735.00 (360.50 to 2,184.00)	701.00 (219.00–2,008.00)	1,023.50 (361.00–2,190.00)	
Graft Survival (days) Median (IQR)	730.00 (324.00–2,170.50)	701.00 (219.00–2,008.00)	996.00 (349.00–2,188.00)	
Post-transplant PTM recurrence				
No	108 (89.26%)	16 (84.2%)	92 (90.2%)	0.711
Yes	13 (10.74%)	3 (15.8%)	10 (9.8%)	
Time from transplant to PTM recurrence (days) Median (IQR)	730.00 (349.00–2,169.00)	701.00 (219.00–1,536.50)	755.00 (360.00–2,172.00)	

PTM, pre-transplant malignancy; ICU, intensive care unit; IQR, inter-quartile range.

### 3.4 Impact of recurrence on survival

There were 20 (14%) patients with known post-LT recurrence of their PTM (Table 4), with median time to recurrence of 596 days (IQR 285–1,415 days). The most frequently recurring malignancies were cutaneous (*n* = 14), colorectal (3), breast (2), and GU (2). Recurrence was significantly more frequent in patients with cutaneous PTM (*p* < 0.001). Other peri-LT variables did not significantly differ between patients that did and did not have a recurrence of their PTM (Table 4). Here we define ‘recurrence-free survival’ as time from transplant to recurrence of the patient's PTM. Recurrence-free survival at 1 year, 3 years, and 5 years was 94.10%, 87.30%, and 81.90% respectively. Time to recurrence was also not associated with peri-transplant variables (Supplementary Table S1B).

**Table 4:** Peri-operative liver transplant variables in patients with and without recurrence of pre-transplant extrahepatic malignancy.

Variable	All patients with PTM	No recurrence of PTM	Recurrence of PTM	*p*
Gender				
Female	65 (45.45%)	59 (48%)	6 (30%)	0.21
Male	78 (54.55%)	64 (52%)	14 (70%)	
Waitlist time (days) Median (IQR)	216.00 (41.00–458.00)	216.00 (39.50–458.00)	204.00 (48.00–477.00)	
Age at transplant (years) Median (IQR)	62.00 (57.00–67.00)	62.00 (57.00–67.00)	60.00 (54.00–68.50)	0.859
BMI (kg/m^2^) Median (IQR)	28.36 (25.40–32.78)	28.36 (25.39–32.78)	28.62 (25.96–32.64)	0.819
Condition				
Home	82 (57.34%)	72 (58.5%)	10 (50%)	0.73
Inpatient non-ICU	21 (14.69%)	18 (14.6%)	3 (15%)	
ICU	40 (27.97%)	33 (26.8%)	7 (35%)	
Etiology of liver failure				
Auto-immune	11 (7.69%)	9 (7.3%)	2 (10%)	0.709
Congenital	8 (5.59%)	6 (4.9%)	2 (10%)	
Metabolic	29 (20.28%)	25 (20.3%)	4 (20%)	
Other	31 (21.68%)	27 (22%)	4 (20%)	
Toxic	31 (21.68%)	29 (23.6%)	2 (10%)	
Viral	33 (23.08%)	27 (22%)	6 (30%)	
MELD at transplant	22.00 (11.00–31.00)	22.00 (11.00–30.50)	28.50 (11.50–37.50)	0.199
Overall survival (days) Median (IQR)	735.00 (360.50–2,184.00)	730.00 (354.50–2,170.50)	1,091.50 (545.50–2,737.50)	
Graft Survival (days) Median (IQR)	730.00 (324.00–2,170.50)	729.00 (309.50–2,170.50)	1,013.00 (324.00–2,144.00)	

PTM, pre-transplant malignancy; ICU, intensive care unit, IQR, inter-quartile range.

Post-LT OS probabilities at 1, 3, and 5 years for patients **without recurrence** were 89.5%, 74.4%, and 74.4%, respectively. Survival probabilities at 1, 3, and 5 years for patients **with recurrence** were 74.4%, 51.8%, and 44.4%, respectively. Recurrence of PTM, when treated as a time-dependent co-variate, was strongly associated with worse post-LT OS (HR 10.9, 95%CI 4.32–27.7, *p* < 0.001) (Supplementary Table S1C; Figure 1D).

Of the 20 patients who experienced recurrence (Supplementary Table S2), 15 were treated with curative intent and 5 palliatively. Treatments included surgical resection in 14 patients, chemotherapy in 7 patients, and radiation therapy in 7 patients. Six patients passed post-recurrence, including 5 who died of recurrence-related events.

## 4 Discussion

The literature describing the outcomes of LT patients with PTM is limited compared to other SOT populations. We have found in our LT population a PTM rate of 7.6%. Our patients with PTM demonstrated similar outcomes to those without PTM. Time less than vs. greater than 2 years between PTM and LT did not affect survival outcomes. Patients with recurrence of their PTM had significantly worsened survival, although risk of recurrence was not associated with pre-transplant variables.

### 4.1 Pre-transplant malignancy rates in liver transplantation

We identified a PTM rate of 7.6% in our patient population which is higher than previously reported by other Korean and Japanese institutions; 2.9% and 4.4% respectively (7, 8). The types of malignancy reported by these institutions differed from those found in our study. We report a significant proportion of patients with skin and GU malignancies. Other studies report a greater proportion of breast, GI, and thyroid malignancy. In comparison to other SOT populations, our PTM rate is comparable to those of HT (7.7%) and KT (6.9%) literature (6, 9).

### 4.2 Liver transplantation in patients with vs. without PTM

In our study, patients with PTM demonstrated similar outcomes to those without PTM. This contrasts with the existing SOT literature. Acuna and colleagues found that in all SOT in Ontario, Canada, from 1991 to 2010, those with PTM had worsened OS (10.3 vs. 13.4 years) and were at increased risk of both cancer-specific (HR 1.85, 95%CI 1.20–2.86) and non-cancer mortality (HR 1.29, 95%CI 1.08–1.54) (10). Similarly, Hart and colleagues studied all U.S. SOT recipient data and found that a history of PTM was associated with increased overall mortality (HR 1.45, 95%CI 1.40–1.50) and cancer-specific mortality (HR 2.73, 95%CI 2.49–2.99) (11). In both the Acuna and Hart studies, the increased post-LT mortality risk was only seen with certain “high risk” PTM. In the kidney transplant literature, PTM is predominately associated with poorer outcomes (9, 12). In the heart transplant literature, there is no consensus at this time with several conflicting studies (6, 13, 14). The improved post-LT outcomes reported here for PTM patients may be due to our focus on only the LT population, only on a more recent time period, or on practice patterns particular to our center informed by more modern guidelines.

### 4.3 Time from PTM to transplant

We found that time less than vs. greater than 2 years from PTM to LT did not affect survival outcomes in our patient population. This contrasts with the existing literature on LT patients with PTM. Park and colleagues studied outcomes of living donor LT (LDLT) recipients with incidentally diagnosed PTM (7). They found that incidentally diagnosed PTM were significantly more likely to recur after transplant than remotely treated PTM (14.6% vs. 3.3%, *p* = 0.025) with a corresponding decrease in OS (*p* = 0.046). A meta-analysis by Acuna and colleagues studying all SOT recipients with PTM found that in LT patients, wait time between PTM to transplant of less than 5 years were at significantly increased risk of recurrence (15). At our institution, wait times are determined on a case-by-case basis via discussion amongst the LT team with particular input from a medical oncologist determining whether the 5-year survival likelihood of the patient is greater than 80%. This may help to produce similar survival outcomes in patients with PTM with less wait time than what is currently recommended by society consensus statements (5). In the broader SOT literature, there is additional evidence to suggest that wait times from PTM to transplant of shorter than the historically-used 5 years are adequate to produce similar outcomes to those without PTM. Dahle and colleagues found in a review of the Norwegian data that while KT with PTM had increased overall cancer mortality (HR 1.97, 95% CI 1.51–2.56), this was not associated with worsened all-cause mortality, cancer-specific mortality, or recurrence. The authors highlighted that the Norwegian policy of a short wait period of 1 year between PTM and KT produced similar survival outcomes compared to those without a history of PTM (12). Youn and colleagues performed a single-institution review of 1,062 HT recipients and did not identify a significant difference in mortality when stratifying by a wait time from PTM to transplant of 5 years (13).

### 4.4 Recurrence of PTM

Recurrence of PTM was strongly associated with poorer outcomes in our study. This is consistent with the existing SOT literature both in the U.S. and abroad. Of note, a meta-analysis by Acuna and colleagues found a pooled cancer recurrence risk of 1.6 per 100 person-year, with LT recipients having the lowest risk of cancer recurrence (1.0 event per 100 person-year) and KT recipients having the highest risk of cancer recurrence (2.4 events per 100 person-year) (15). In patients with PTM, immunosuppression may increase risk of cancer recurrence. Small bowel/multi-visceral transplantation requires the greatest amount of immunosuppression, followed in decreasing order by lung, heart, pancreas, kidney and liver transplant (16). The liver demonstrates immunological tolerance, has lower frequency of rejection compared to other SOT, and LT patients can typically tolerate a lower degree of immunosuppression (17). The decreased immunosuppression requirement of LT compared to other SOTs may contribute to the lower cancer recurrence risk seen in LT (18). We did not identify pre-LT variables associated with recurrence, and unfortunately recurrence was associated with decreased post-LT survival, consistent with the literature.

### 4.5 Limitations

Our study included only LT patients from a single U.S. center, and thus is smaller in sample size than pan-SOT national studies previously reported. This offers a greater degree of granularity and reliability. Nonetheless, details on PTM histology and treatment were not always available for review. “Recurrence” was defined anatomically, rather than histologically or molecularly, likely overestimating the true PTM recurrence rate. For example, many reported recurrences were cutaneous malignancies, and it is difficult to ascertain retrospectively from health records whether these were true recurrences vs. *de novo* malignancies which are common in immunosuppressed patients. Furthermore histopathological diagnosis was often unspecified for cutaneous malignancies thus preventing delineation between melanomatous and non-melanomatous cancers.

## 5 Conclusions

To our knowledge, this is the largest single center study of LT patients with PTM. There is a need for more data on this subject in the literature, as decision-making is currently informed by limited outcome data from both the general SOT population and HT and KT patient subsets. We report that a history of PTM alone does not worsen post-LT survival, nor do shorter wait times of less than 2 years. This suggests that our institutional practice of screening patients with PTM history based on an estimated 80% 5-year survival is sufficient to produce comparable outcomes to LT patients without PTM. Additional research is needed to determine whether immunosuppression intensity in the LT population has a protective role. Recurrence of PTM after LT is associated with significantly worse survival, but unfortunately could not be predicted using pre-LT variables.

## Data Availability

The raw data supporting the conclusions of this article will be made available by the authors, without undue reservation.
